# Regulatory approach to manipulated organs in Europe: preserving human organs as non-commercial goods 

**DOI:** 10.3389/ti.2026.16447

**Published:** 2026-06-16

**Authors:** Natividad Cuende, Ander Izeta, Beatriz Domínguez-Gil

**Affiliations:** 1 Andalusian Transplant Coordination, Andalusian Health Service, Seville, Spain; 2 Hospital Universitario de Donostia, Donostia-San Sebastian, Spain; 3 Biogipuzkoa Health Research Institute, Donostia-San Sebastian, Spain; 4 Organizacion Nacional de Trasplantes, Madrid, Spain

**Keywords:** advanced therapy medicinal products (ATMPs), equitable access, genetically modified human organs, legal framework, organ donation and transplantation, medical devices, *ex vivo* organ perfusion, substances of human origin (SoHO)

## Abstract

We welcome the European Commission’s initiative to amend the European Organ Directive within the framework of the proposed Biotech Act, intended to align it with recent scientific and technological advances, particularly organ manipulations aimed at improving transplant outcomes and human health. However, the amendment introduces a definition of organ processing that raises significant regulatory questions, particularly related to the exclusions it contains, which might allow human organs subjected to genetic or enzymatic manipulation to be regulated as medicinal products or as goods under industrial or commercial legal frameworks. The potential consequences of regulating human organs as medicines are reviewed, and awareness is raised of the risk of undermining the altruistic basis of donation and public trust in organ donation and transplantation systems. Moreover, this shift could jeopardise access to this life-saving therapy and threaten the economic sustainability of healthcare systems. Concurrently, potential risks associated with certain forms of manipulation (e.g., genetic modification of organs) must be rigorously assessed before their incorporation into clinical practice. We propose a distinctly European regulatory model that preserves the intrinsic value of human organs, promotes collaboration between medicines and transplant authorities, and enables biomedical innovation through companies providing services and technical solutions to healthcare systems.

## Introduction

We are witnessing extraordinary scientific and technological advances in organ transplantation aimed at increasing the availability of organs for clinical use [1] and improving their functionality. Newly developed techniques, such as enzymatic [2] or genetic [3] manipulations, may optimise transplant results and improve health outcomes [4, 5]. Most of these developments are enabled by novel *ex vivo* organ perfusion techniques [6, 7]. Overall, these advances offer new hope for patients in need but also pose significant challenges for healthcare systems and for the current model of organ donation and transplantation.

The transplantation of manipulated human organs presents additional biological risks beyond those associated with standard procedures. Before modified organs can be incorporated into clinical practice, such risks must be carefully characterised and minimised. Once quality, safety, functionality and efficacy are established, incorporation into healthcare services will require integration within existing organ donation and transplantation programmes, supported by strengthened biovigilance and long-term follow-up systems.

On 16 December 2025, the European Commission (EC) made public its proposal for a Biotech Act [8], aimed at enhancing the competitiveness of European biotechnology companies in the healthcare sector, together with a proposed Directive [9] amending two existing Directives, including Directive 2010/53/EU (hereinafter, the Organ Directive) [10] on the quality and safety of human organs intended for transplantation (xenotransplantation falls outside the scope of the Directive).

We welcome the initiative to amend the Organ Directive, as it enables the legislation to adapt to scientific and technological advances. However, it is concerning that the proposed amendment might allow human organs subjected to specific manipulations to fall outside the scope of the Organ Directive or to be regulated under legal frameworks designed for marketable goods, thereby introducing a significant risk of commodification of human organs, with ethical and social consequences that could undermine organ donation and transplantation systems.

This manuscript analyses the regulatory implications of the proposed amendment to the Organ Directive for modified human organs, as well as its potential consequences, and seeks to contribute to the development of a European model that reconciles biomedical innovation with the protection of the intrinsic value of human organs.

## An ambiguous regulatory framework may allow the commodification of human organs

### Main amendment introduced by the revision of the Organ Directive

The original scope of the Organ Directive was “the donation, testing, characterisation, procurement, preservation, transport and transplantation of organs intended for transplantation” [10]. In the amended Directive, the term “preservation” is replaced by “processing”, and “organ processing” is defined as “operations involving the handling of organs, including but not limited to preservation, application of chemotherapy and surgery, performed to maintain or improve organ function prior to transplantation”. The definition of processing excludes the following: (1) “the preparatory handling of the organ during the surgical transplantation intervention”; (2) “the repurposing of organs into tissues or cells”; and (3) “the use of a substance with a pharmacological, immunological or metabolic action with the aim to treat or prevent a disease in the recipient to whom the organ will be transplanted, where such use does not constitute processing of the organ”.

Of note, the definition of a medicinal product in Directive 2001/83/EC (which currently regulates medicinal products in the European Union [EU]) [11] is very similar in wording to the third exclusion set out in the definition of organ processing. It is therefore reasonable to argue that medicinal products may be used in donated organs without this constituting organ processing.

As a consequence, under the proposed amendment, manipulations performed on human organs prior to transplantation through treatment with medicinal products—for example, to induce genetic or enzymatic modifications—would not meet the definition of organ processing and would therefore fall outside the scope of the Organ Directive. In such cases, organs subjected to these interventions might instead be regulated as medicinal products or, more broadly, as commercial or industrial goods.

Moreover, even if the processing of a human organ through the use of a medicinal product or another type of product specifically developed for that purpose were considered to fall within the regulatory framework of the Organ Directive, the proposed amendment to the Organ Directive, which establishes a requirement to authorise such products under their respective regulatory frameworks, would in effect entail subjecting manipulated human organs to regulatory regimes designed for marketable goods.

Understanding the basis for and consequences of this potential regulatory shift requires consideration of the recent activity of the Committee for Advanced Therapies (CAT) of the European Medicines Agency (EMA) immediately preceding the publication of the proposal to amend the Directive. It also requires consideration of the differences between the regulatory pathways governing clinical research and the authorisation of medicines and transplants, and of the paradigm-shifting distinction between access to medicines and organ allocation.

### Classification of viral vectors used to genetically modify human organs as medicinal products

On 7 November 2025, the CAT recommended that a viral vector intended to modify human lungs *ex vivo* prior to transplantation be classified as an advanced therapy medicinal product (ATMP), specifically as a gene therapy medicinal product (GTMP). The vector incorporates a sequence to silence HLA antigen expression in donor organs and mitigate immune-mediated rejection in transplant recipients.

The CAT stated that “This classification is without prejudice to the status of the genetically modified organ of human origin and the GMO legislation.” [12] In other words, the CAT refrained from taking a position on the classification of the genetically modified organ itself, since the current European legal framework does not envisage the qualification of human organs as medicinal products [11, 13, 14] or as products distinct from human organs. However, a few weeks after the CAT issued this recommendation, the EC published its proposal to amend the Organ Directive [9] within the context of the proposed Biotech Act [7].

Based on the definition proposed by the EC, the use of a viral vector (classified as a medicinal product) to genetically modify human lungs in order to reduce immunogenicity and prevent graft rejection would not be considered organ processing, and the genetically modified lungs would therefore fall outside the scope of the Organ Directive. We may thus be moving from speculation to reality, as the amended text opens the door to genetically or enzymatically modified human organs being regulated under alternative legal frameworks.

### Regulatory ambiguity

Even though genetically modified organs are not excluded from the scope of the Organ Directive nor expressly regulated as medicinal products, the requirement introduced by the revised Organ Directive to authorise medicinal products under their respective regulatory frameworks prior to the authorisation of the processing of human organs generates regulatory ambiguity and might result in manipulated human organs being regulated under the legal framework for medicines.

The vector used to modify the organ is a relevant example. It has itself been classified as a medicinal product, yet its effects can only be assessed in the transplanted organ. Therefore, evidence of safety and efficacy (essential requirements for marketing authorisation) would necessarily be generated in clinical trials in which the transplantation of the modified organ is evaluated. In practice, this would mean that the modified organ would fall under the regulatory framework applicable to investigational medicinal products. Nevertheless, in order to conduct such clinical trials, altruistically donated human organs used for genetic modification might ultimately be diverted from transplantation to patients on the waiting list. Moreover, the eligibility criteria for patients to participate in the clinical trial would interfere with the allocation criteria established by the competent transplant authorities.

More importantly, if the quality, safety and efficacy of the genetically modified organ have been demonstrated under the medicines framework, on what basis could marketing authorisation for the vector as a medicinal product be granted if it is not intrinsically linked to the modified organ, which constitutes the actual therapy evaluated in clinical trials? This uncertainty extends to approval pathways and competent authorities, since the processes for incorporating transplants and medicinal products into healthcare systems differ substantially and would be at odds with organ donation and transplantation systems, as discussed below.

This outcome would only be avoided if genetically modified organs are maintained within the scope of the Organ Directive, accompanied by a clear regulatory framework under which the authorisation of organ modifications and their clinical evaluation remain within the competence of the relevant transplant authorities.

Consequently, the classification of a viral vector applied *ex vivo* to human organs as a GTMP creates uncertainty regarding the applicable regulatory framework for assessing the quality, safety and efficacy of the therapy, which, in effect, is the genetically modified organ and does not itself qualify as a medicinal product, thereby creating a conflict between regulatory frameworks.

It is possible that the CAT’s recommendation to classify the viral vector as a GTMP was influenced by concerns about the biological risks associated with genetic modifications of human organs. At the time of classification, no proposal to amend the Organ Directive—potentially allowing genetically modified organs to be regulated as medicines—had been published. This may have contributed to classifying the viral vector as a medicinal product to ensure some degree of regulatory oversight of genetically modified organs. Nevertheless, in our view, the viral vector does not fully meet the definition of GTMP.

### Some aspects to consider on the classification

On the current EU legal framework [14], “Gene therapy medicinal product means a biological medicinal product which has the following characteristics: (a) it contains an active substance which contains or consists of a recombinant nucleic acid used in or administered to human beings with a view to regulating, repairing, replacing, adding or deleting a genetic sequence; (b) its therapeutic, prophylactic or diagnostic effect relates directly to the recombinant nucleic acid sequence it contains, or to the product of genetic expression of this sequence.”

First, the viral vector is neither used in nor administered to human beings. It is applied to human organs for *ex vivo* modification before transplantation. Administration is therefore indirect, analogous to *ex vivo* gene therapy, where a viral vector modifies human cells before administration to the patient. In such cases, the genetically modified cells (rather than the viral vector itself) constitute the GTMP.

Second, although the CAT states that “its therapeutic effect relates directly to the product of genetic expression of this sequence”, the therapeutic effect derives from the transplanted organ, not from the viral vector, which merely mitigates the risk of rejection. The genetic modification of the organ improves durability rather than constituting the therapy.

While the classification of a vector for *ex vivo* human organ modification as a GTMP could be viewed by some as irrelevant, we take a different view: it implies that genetically modified human organs would fall under the investigational medicinal products framework—thereby interfering with organ donation and transplantation rules—and would open the door to their healthcare provision being delivered by pharmaceutical and biotech companies holding marketing authorisation. This would represent a seismic shift in the way organ transplantation is currently implemented in Europe.

## Consequences of regulating human organs as medicinal products

There is no doubt that human organs genetically modified *ex vivo* prior to transplantation pose higher biological risks associated with the genetic modification. Assessment of these risks, as well as evaluation of the modified organ’s quality, safety, functionality and effectiveness, must be conducted according to strict standards proportionate to the level of risk. However, classifying the vector as a medicinal product, and potentially manipulated organs as commercial goods, entails significant implications. In particular, it remains unclear how such therapies would be integrated into existing organ donation and transplantation models grounded in altruistic donation, non-remuneration of organs and equity of access.

If organs—or vectors used for their genetic modification—are regulated as ATMPs, the altruistic, not-for-profit model risks shifting towards a commodity-based framework in which patient access depends on for-profit entities obtaining marketing approval, followed by price negotiations, reimbursement decisions and commercial strategies. The potential consequences, grouped below into three categories, may compromise altruistic donation and undermine the current organ donation and transplantation systems.

### Difficulties in patient accessibility

Experience with the regulatory framework for human cells and tissues provides relevant lessons [7, 15]. Following adoption of Regulation (EC) No 1394/2007 on ATMPs [13], certain therapies previously regulated as transplants migrated to the medicines framework, including artificial skin transplants, limbal cell and chondrocyte cultures. Patient access became conditional upon centralised marketing authorisation or, alternatively, authorisation under the hospital exemption (HE) scheme [16].

Nearly two decades after approval of the ATMP Regulation, no company in the EU has commercialised artificial skin, which represents the last therapeutic option for severe burns. One company obtained centralised marketing authorisation for autologous limbal stem cells (HOLOCLAR®), yet access across Member States has remained limited due to lack of reimbursement, and the company required public rescue funding in 2023 shortly before filing for bankruptcy [17]. Three companies obtained marketing authorisation for autologous cultured chondrocyte based products (CHONDROCELECT®, MACI® and SPHEROX®), but two were subsequently withdrawn from the market [18]. At present, the remaining product has limited availability, restricted to a small number of Member States [19], with even fewer providing public funding [20]. The causes of this situation are multifactorial. Among other factors, cost–utility evaluations have often been unfavourable, since the regulation of these therapies as medicinal products increases costs, while clinical efficacy remains unchanged. Consequently, the transition from transplant to ATMP legal framework has increased healthcare expenditure and resulted in limited and unequal access to these therapies [21].

**FIGURE 1:**
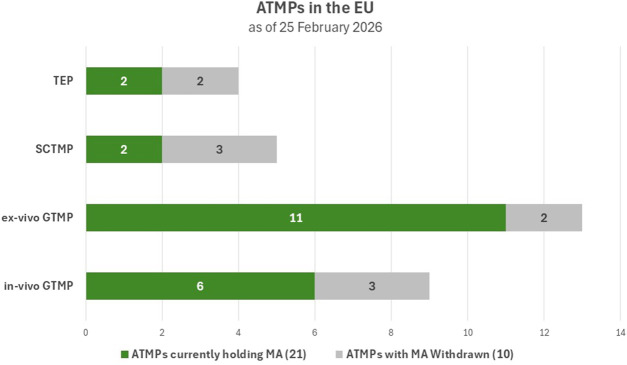
Holding and withdrawal of marketing authorisation (MA) of all Advanced Therapy Medicinal Products (ATMPs) that have been marketed in the EU since the ATMP Regulation was approved (2007) until February 25, 2026, by medicine type. TEP, tissue engineered product. SCTMP, somatic cell therapy medicinal product. GTMP, gene therapy medicinal product. Data source: information curated from the European Medicines Agency website by the authors [17].

More broadly, of the 31 ATMPs granted marketing authorisation in the EU to date, one third have been withdrawn from the market, mainly for commercial reasons following decisions by marketing authorisation holders [18] (Table 1; Figure 1).

**TABLE 1:** Advanced therapy medicinal products (ATMPs) holding marketing authorisation (MA) or with MA withdrawn in the European Union as of 25 February 2026.

ATMP type	Currently holding MA	MA withdrawn
Tissue-engineered products	SPHEROX® HOLOCLAR®	MACI® CHONDROCELECT®
Somatic cell therapy medicinal products	ZEMCELPRO® EBVALLO®	ALOFISEL® ZALMOXIS® PROVENGE®
*Ex vivo* gene therapy medicinal products	WASKYRA® AUCATZYL® CASGEVY® CARVYKTI® BREYANZI® ABECMA® LIBMELDY® TECARTUS® YESCARTA® KYMRIAH® STRIMVELIS®	ZYNTEGLO® SKYSONA®
*In vivo* gene therapy medicinal products	VYJUVEK® HEMGENIX® UPSTAZA® ZOLGENSMA® LUXTURNA® IMLYGIC®	BEQVEZ® GLYBERA® ROCTAVIAN®
Additional information	ATMPs currently holding MA: https://www.ema.europa.eu/en/medicines/human/EPAR/spherox https://www.ema.europa.eu/en/medicines/human/EPAR/holoclar https://www.ema.europa.eu/en/medicines/human/EPAR/zemcelpro https://www.ema.europa.eu/en/medicines/human/EPAR/ebvallo https://www.ema.europa.eu/en/medicines/human/EPAR/waskyra https://www.ema.europa.eu/en/medicines/human/EPAR/aucatzyl https://www.ema.europa.eu/en/medicines/human/EPAR/casgevy https://www.ema.europa.eu/en/medicines/human/EPAR/carvykti https://www.ema.europa.eu/en/medicines/human/EPAR/breyanzi https://www.ema.europa.eu/en/medicines/human/EPAR/abecma https://www.ema.europa.eu/en/medicines/human/EPAR/libmeldy https://www.ema.europa.eu/en/medicines/human/EPAR/tecartus https://www.ema.europa.eu/en/medicines/human/EPAR/yescarta https://www.ema.europa.eu/en/medicines/human/EPAR/kymriah https://www.ema.europa.eu/en/medicines/human/EPAR/strimvelis https://www.ema.europa.eu/en/medicines/human/EPAR/vyjuvek https://www.ema.europa.eu/en/medicines/human/EPAR/hemgenix https://www.ema.europa.eu/en/medicines/human/EPAR/upstaza https://www.ema.europa.eu/en/medicines/human/EPAR/zolgensma https://www.ema.europa.eu/en/medicines/human/EPAR/luxturna https://www.ema.europa.eu/en/medicines/human/EPAR/imlygic ATMPs with MA withdrawn: https://www.ema.europa.eu/en/medicines/human/EPAR/maci https://www.ema.europa.eu/en/medicines/human/EPAR/chondrocelect https://www.ema.europa.eu/en/medicines/human/EPAR/alofisel https://www.ema.europa.eu/en/medicines/human/EPAR/zalmoxis https://www.ema.europa.eu/en/medicines/human/EPAR/provenge https://www.ema.europa.eu/en/medicines/human/EPAR/zynteglo https://www.ema.europa.eu/en/medicines/human/EPAR/skysona https://www.ema.europa.eu/en/medicines/human/EPAR/beqvez https://www.ema.europa.eu/en/medicines/human/EPAR/glybera https://www.biomarin.com/news/company-statements/biomarin-voluntarily-withdraws-roctavian-from-the-market/	

Notably, accessibility to these therapies not only depends on marketing authorisation but also on drug availability and public reimbursement. Many ATMPs are available in only a few EU Member States, partly due to companies’ limited manufacturing and supply capacities, which lead them to prioritise markets based on commercial strategy, market size, and national pricing and reimbursement systems [19, 20]. Reimbursement for approved indications also varies considerably, with some countries funding only narrower indications than those authorised and delaying decisions to contain pharmaceutical expenditure [22].

The HE scheme, applicable under specific conditions, has also not ensured equitable access across Europe. An EC report [23] showed considerable variability in national implementation. By 2025, over 40% of Member States had not authorised treatments under HE schemes, often due to the absence of national legislation. Among those that had done so, both the number of patients and review timelines varied substantially [24].

In summary, access to ATMPs remains limited and uneven across the EU. This experience suggests that the transition from the transplant to the medicines regulation has not favoured the development of, or equitable access to, these therapies [25].

### Risks to the economic sustainability of healthcare systems

The negotiated price of commercial ATMPs ranges from tens or hundreds of thousands of euros to several million euros per dose [19, 20]. Such pricing poses a clear challenge to the sustainability of publicly funded healthcare systems and contributes to restricted access [7]. Beyond the negotiating leverage of individual countries, the factors underlying drug pricing are complex, depending partially on manufacturing costs and legitimate profit expectations. Additionally, the medicines framework, compared with the transplant framework, entails further costs, including those for obtaining marketing authorisation and fulfilling post-authorisation obligations.

For example, the production of the GTMP STRIMVELIS®—the only curative treatment for ‘bubble children’—initially marketed by two pharmaceutical companies and later ‘rescued’ by a public institution, involves substantial fixed costs, 25% of which are regulatory [26]. Fees payable to the EMA, already burdensome for public institutions, have recently risen [27]. These factors threaten continued market availability, particularly for ultra-rare conditions [28].

Experience with human cells and tissues [25] should therefore serve as a cautionary precedent [29]. Regulating manipulated human organs within the medicines framework may replicate similar challenges for sustainability and access.

### Ethical conflicts

Regulating modified human organs as medicines and subjecting them to market forces would entail substantial ethical implications. The donation and transplantation of human organs, given their unique origin, are founded on ethical principles promoted by the World Health Organisation [30–32], the Council of Europe [33–35] and the EU [10, 36]: non-commercialization of the human body, voluntary and unpaid donation, and allocation governed by a transparent and fair system, guided by clinical criteria and ethical norms. These principles underpin public trust and participation in organ donation.

The principle of non-commercialisation of the human body is enshrined in the EU Charter of Fundamental Rights, which prohibits “making the human body and its parts, as such, a source of financial gain” [36]. Regulating modified organs as medicines would give rise to a marketable product capable of generating economic benefit for marketing authorisation holders—profit not subject to regulation beyond market rules—, potentially violating such fundamental principle [37].

Commercialisation may also undermine altruistic donation. Public perception that donated organs generate significant economic benefit for private entities could erode trust in transplant systems [15, 38]. Such commercialisation would pose a serious risk to donation and transplantation programmes in the EU, which rely entirely on altruistic donation and on a uniquely valuable yet highly vulnerable asset: public trust in a system governed by principles fundamentally different from those of the market. Any erosion of that trust could reduce willingness to donate and thereby jeopardise access to life-saving transplantation for thousands of patients.

Furthermore, Europe’s commitment to equitable access requires that modified organs be fully integrated into the same allocation and prioritisation rules as unmodified organs, based solely on clinical criteria to ensure equitable access. The coexistence of dual pathways for organ availability—donated and acquired organs—would threaten such integration.

If modified organs were commercialised and not publicly funded, or reimbursed only for restricted indications—as frequently occurs with ATMPs—this could not only create inequities in access but also undermine donation and transplantation programmes for organs and other substances of human origin (SoHO). Moreover, if wealthy individuals were able to gain access to transplantation of a modified organ by paying for it, this would constitute a direct and irreversible threat, since altruistic donation depends not only on trust but also on the principle of reciprocity. Ultimately, the mere prospect that an altruistically donated, subsequently modified organ could be commercialised and acquired by those with high purchasing power would signal the end of donation and transplantation programmes.

## How to promote technological innovation while preserving the ethical principles governing the donation and transplantation of modified organs

Our proposal is grounded in the premise that human organs intended for transplantation—regardless of any manipulation or clinical indication—must remain regulated within the transplant framework and protected from commodification. Table 2 presents a proposal for a governance model to integrate manipulated organs into the current organ donation and transplantation systems and highlights potential ethical issues that may arise.

**TABLE 2:** Proposed governance model to integrate manipulated organs on the current organ donation and transplantation systems and potential ethical issues arising.

Step	Who does it	Who oversees/Evaluates it	Who authorizes it	Who owns it	Potential ethical issues
Organ donation (identification, characterization, evaluation, maintenance, consent, authorization, recovery, preservation)	Healthcare systems	Transplant organizations	Transplant authorities	Organs are owned by the society at large	Need to ensure donation remains voluntary and unpaid, to respect human dignity and integrity and to ensure the quality and safety of donated organs
Manipulation of human organs intended for transplantation	Companies act as service providers	Medicines agencies in collaboration with transplant organizations, using standards based on pharmaceutical legislation	Transplant authorities and ethics committees	Companies own the technology	Risk of diversion of transplantable organs if scientific and ethical criteria are not clearly predefined Inequities in access to manipulated organs due to unaffordable service fees
Non-clinical research and quality characterization of manipulated human organs			Ethics committees		
Clinical research and assessment of safety, functionality and effectiveness of manipulated human organs	Healthcare systems in collaboration with companies	Transplant organizations in collaboration with medicines agencies	Transplant authorities and ethics committees	Manipulated organs are owned by the society at large	Differing attributes and clinical outcomes between manipulated and non-manipulated human organs may affect decision-making by transplant organizations and affect prioritization
Allocation of manipulated human organs	Healthcare systems	Transplant organizations, with manipulated organs being integrated into the transplant prioritization system	Transplant authorities		
Transplantation of manipulated human organs and long-term monitoring of outcomes		Transplant organizations, with transplants performed in specifically authorized centres			Need to ensure equitable access to avoid potential family refusal of organ donation

### Industry’s role and organ versus technology ownership

Innovation in organ transplantation requires industry participation. However, such involvement must not compromise the ethical standards governing donation and transplantation [30–36]. Within the medicines framework, companies hold ownership of marketed products. This conflicts with the European transplant model, where organs are altruistically donated to society and allocated through public systems. Companies may legitimately own the technologies used for organ manipulation, but not the organs themselves.

Under the transplant regulatory framework, companies could instead act as service providers for organ modifications, subject to transparent oversight consistent with donation and transplant principles. Although this could seem commercially less attractive than full product ownership, this model would offer advantages: simpler and faster integration into healthcare systems [39], lower regulatory fees and broader uptake. Experience has shown that the high prices of ATMPs lead to restrictions in publicly funded treatments and that, where funding is granted, it is often limited to narrower indications than those authorised.

### Collaboration among relevant authorities and distinction between assessment and authorisation procedures

Legitimate concerns regarding biological risks must be addressed through rigorous evaluation. However, assessment and authorisation are distinct processes. Authorising genetically modified organs as transplants does not preclude prior evaluation applying standards inspired by the pharmaceutical legislation, nor does it exclude collaboration between medicines agencies and transplant authorities.

In some Member States, such cooperation already exists. For example, medicines authorities may require that transplant organisations assess donor evaluation and organ recovery before authorising clinical trials involving ATMPs derived from human tissues or cells. Similar reciprocal collaborative mechanisms could be established to ensure that manipulated human organs undergo comprehensive assessment of quality, safety, preclinical evidence, patient follow-up and clinical outcomes prior to authorisation under the transplant framework.

Alternatively, transplant organisations could develop strengthened methodologies for evaluating innovation in organ transplantation. Such an approach is already applied to other SoHO [40], in accordance with the relevant European Regulation [41].

### Reinforcing the Organ Directive

The draft Biotech Act and the proposed amendment to the Organ Directive represent an opportunity to establish a robust European legal framework governing transplantation of manipulated organs while preserving ethical principles already enshrined in the current Organ Directive.

However, we strongly advocate the revision of the proposed definition of organ processing to encompass any form of manipulation to which human organs may be subjected. Furthermore, to ensure equitable access, the Organ Directive should also require Member States to maintain transparent allocation systems guided by clinical criteria and ethical standards.

The Organ Directive should strengthen evaluation and biovigilance mechanisms, while ensuring that clinical research on organ modifications and their authorisation remain within the competence of the relevant transplant authorities, in line with the European SoHO Regulation [41], which offers clear advantages over the ATMP Regulation: lower costs; greater flexibility in collecting safety and clinical outcome data based on risk assessment; authorisation of SoHO preparations by national competent authorities under a harmonised EU procedure; a suitable biovigilance system; more equitable and timely access; and a regulatory framework that upholds the principle of non-commercialisation of the human body [39].

Additionally, follow-up registries of living donors and transplant recipients should be explicitly required, as they are essential for assessing the safety and effectiveness of organ transplantation and processing outcomes. Likewise, in the public health interest, competent authorities must have access to appropriate data, in full compliance with EU data protection rules, to ensure the safety and effectiveness of donation and transplantation processes and to enable international information exchange.

### Integration of manipulated organs into donation and transplantation systems

Two key elements are essential for proper integration of manipulated organs into donation and transplantation systems without compromising their fundamental principles.

First, donor selection for organs intended for manipulation must follow scientific and ethical criteria approved by competent transplant authorities. The donation process must remain fully integrated within official programmes, under the responsibility of donor coordinators or procurement organisations, who oversee donor identification, evaluation, and maintenance, as well as coordinate all activities from donation to transplantation, in accordance with protocols approved by transplant authorities.

Second, manipulated organs must be incorporated into the organ allocation system. Accordingly, prioritisation of patients requires that all those eligible for transplantation be registered on the official waiting lists under the supervision of the competent transplant authorities. Both the clinical criteria for inclusion on waiting lists and those governing allocation of different types of organs should be established under the oversight of the competent authorities to ensure equitable access.

Regulating manipulated organs as transplants rather than medicinal products would contribute to healthcare economic sustainability while strengthening the donation model based on altruism, free provision and equitable access. Preserving this model is essential, as it saves thousands of lives annually and improves quality of life for many more.

## Conclusion

We advocate a genuinely European model in which biomedical innovation progresses in alignment with the protection of the intrinsic value of human organs, supporting technological developments as services and technical solutions, while ensuring that human organs—regardless of manipulation or therapeutic/preventive use—remain outside any profit-driven commercial framework. This model is consistent with the ESOT position paper on ATMPs in the context of transplantation [7], which offers strategic recommendations to facilitate access to these technologies across Europe and aims to improve transplant outcomes. Its first recommendation highlights the need to streamline regulatory procedures to expedite approval timelines and reduce associated costs, while maintaining stringent standards of safety and efficacy.

Only under such an approach can scientific and clinical excellence advance without compromising the values that have made Europe’s donation and transplantation systems a global benchmark. The Biotech Act and the proposed amendment to the European Organ Directive—provided they preserve the altruistic, non-commercial nature of human organs—offer a strategic opportunity to establish a robust framework for organ manipulation, promoting technical and scientific progress without abandoning the ethical principles underpinning Europe’s moral leadership in transplantation.
